# Investigation of the Prevalence of Antibiotic Resistance Genes According to the Wastewater Treatment Scale Using Metagenomic Analysis

**DOI:** 10.3390/antibiotics10020188

**Published:** 2021-02-15

**Authors:** Keunje Yoo, Gihan Lee

**Affiliations:** Department of Environmental Engineering, Korea Maritime and Ocean University, Busan 49112, Korea; dlrlgks77@naver.com

**Keywords:** antibiotic resistance gene, mobile genetic elements, wastewater treatment plant, wastewater treatment scale, metagenomics

## Abstract

Although extensive efforts have been made to investigate the dynamics of the occurrence and abundance of antibiotic resistance genes (ARGs) in wastewater treatment plants (WWTPs), understanding the acquisition of antibiotic resistance based on the WWTP scale and the potential effects on WWTPs is of relatively less interest. In this study, metagenomic analysis was carried out to investigate whether the WWTP scale could be affected by the prevalence and persistence of ARGs and mobile genetic elements (MGEs). As a result, 152 ARG subtypes were identified in small-scale WWTP samples, while 234 ARG subtypes were identified in large-scale WWTP samples. Among the detectable ARGs, multidrug, MLS (macrolide–lincosamide–streptogramin), sulfonamide, and tetracycline resistance genes had the highest abundance, and large and small WWTPs had similar composition characteristics of ARGs. In MGE analysis, plasmids and integrons were 1.5–2.0-fold more abundant in large-scale WWTPs than in small-scale WWTPs. The profile of bacteria at the phylum level showed that Proteobacteria and Actinobacteria were the most dominant bacteria, representing approximately 70% across large- and small-scale WWTPs. Overall, the results of this study elucidate the different abundances and dissemination of ARGs between large- and small-scale WWTPs, which facilitates the development of next-generation engineered wastewater treatment systems.

## 1. Introduction

The WHO has announced that the rapid development of the spread of antibiotic resistance is one of the top 10 global public health threats facing humanity [1]. Due to the overuse and misuse of antibiotics in human activities such as agriculture, livestock, and medical treatment, antibiotic resistance has rapidly accelerated in environmental ecosystems [2,3]. Aquatic environments are regarded as a major source for the aggregation and dissemination of antibiotic resistance genes (ARGs) because they are easily influenced by human activities [4,5]. In particular, the emergence of ARGs could significantly increase the spread of antibiotic resistance, especially via mobile genetic elements (MGEs) including plasmids, integrons and insertion sequences [4,5,6]. There is growing evidence that wastewater treatment plants (WWTPs) have received special attention in playing a major role in the proliferation of antibiotic resistance into other environmental systems, such as rivers, sediment, and oceans [6]. Because WWTPs are mainly designed for the removal of physical and chemical pollutants, ARGs and antibiotic-resistant bacteria (ARB) are usually not considered for the effective removal of antibiotics in WWTPs. Therefore, WWTPs can be a reservoir and vehicle for the transmission of biological contaminants such as pathogenic bacteria and antibiotic resistance mechanisms to other environmental media [7].

Recent studies have consistently reported relatively high levels of ARGs and MGEs and even heavy metal resistance genes in WWTPs and in each process in wastewater treatment systems [6,8,9]. However, most previous studies preliminarily focused on the identification of ARGs and ARB during the wastewater treatment process, such as activated sludge and anaerobic digestion processes, not considering the scale and treatment capacity of WWTPs [6,8]. According to a previous study, the capacity of a WWTP can affect the bacterial community via various operation practices, such as influent characteristics, sludge retention time (SRT), hydraulic retention time (HRT), reactor volumes, and physical constraints [10]. Recent studies have suggested that the bacterial community plays a key role in ARG prevalence and enrichment during the WWTP process [11]. In addition, coselection of the bacterial community and ARGs is favored during the wastewater treatment process due to the massive amount of nutrient substances and certain operation conditions [12].

Although extensive efforts have been made to investigate the dynamics of the occurrences and abundances of ARGs in the activated sludge process using omics approaches, such as metagenomics, metatranscriptomics, metaproteomics, and real-time qPCR approaches [11,13,14,15], the understanding of antibiotic resistance and MGE contamination according to WWTP capacity is understudied. In addition, it is still unclear to what extent water-borne ARGs promote the acquisition of antibiotic resistance among bacterial communities via MGEs according to the WWTP scale. Therefore, this study aimed to investigate whether the WWTP scale could be affected by the prevalence and persistence of ARGs and MGEs. For this, shotgun metagenomics was applied because it is a good approach to gain deeper insights into the entire genomic pool from the mixed environmental microbiome [16,17,18].

## 2. Materials and Method

### 2.1. Study Area and Sampling

Twelve samples (influent, activated sludge, effluent) in this study were collected and two duplicate biological replicate samples were considered for each sampling point from 4 full-scale WWTPs in Busan city, South Korea, in April 2020. The designed A and B WWTP scales were 9.5 × 10^4^ m^3^/d and 4.0 × 10^4^ m^3^/d, respectively, while the C and D WWTP scales were designed to be 34.0 × 10^4^ m^3^/d and 45.2 × 10^4^ m^3^/d, respectively. Detailed information on the investigated WWTPs is provided in Table 1. Briefly, all WWTPs were activated sludge processes equipped with a series of treatment units, including coagulation, sedimentation, sand filtration, and disinfection. The major difference between the small and large WWTPs is that small WWTPs use the conventional activated sludge process, which includes the following treatment units: primary settling tanks, modified Ludzack–Ettinger (MLE) tanks and secondary settling tanks. However, large WWTPs consist of an activated sludge process with the same treatment method as small WWTPs and a membrane bioreactor (MBR) system using microfiltration (MF) membrane units. The MLE process was designed for the treatment of wastewater mainly from municipal sources with a total design capacity of 27.5–34.5 × 10^4^ m^3^/d and MF membranes of 6.5–10.0 × 10^4^ m^3^/d. All samples were stored at −80 °C in a refrigerator until DNA extraction.

### 2.2. DNA Extraction and High-Throughput Shot-Gun Sequencing

Total genomic DNA was extracted using the FastDNA Spin Kit for Soil (MP Biomedicals, CA, USA) according to the manufacturer’s suggested protocol. Extracted DNA concentrations and purities were measured using a 1% gel and a Nano300 Micro Spectrophotometer (Allsheng, HangZhou, China). Approximately, 5 μg of each purified DNA sample was used for shotgun paired-end library construction. Briefly, DNA fragments were end-polished, A-tailed, and ligated with the full-length adaptor for Illumina sequencing with further PCR amplification and purification. The insert sizes of libraries were analyzed using an Agilent 2100 bioanalyzer (Agilent Technologies, Palo Alto, CA, USA). Shotgun metagenome sequencing was performed by Macrogen (Seoul, Korea) using the TruSeq DNA PCR-Free Kit (Illumina Inc., San Diego, CA, USA) according to the library protocol, where index coding had been added. Each sample was barcoded and analyzed using a 2 × 101 bp paired-end protocol with an Illumina HiSeq 2000 platform. The total output of the 12 metagenome samples was approximately 120 Gb. All metagenomes generated in this study are publicly available via MG-RAST under sample IDs mgs821935, mgs821941, mgs821947, mgs821953, mgs821959, mgs821965, mgs821971, mgs821977, mgs821938, mgs821989, mgs821995, and mgs822001.

### 2.3. Bioinformatics Analysis

Raw sequences from the Illumina HiSeq were trimmed with respect to adapters and low-quality nucleotide stretches using Trimmomatic (version 0.39) [19] with default settings. Trimmed and filtered sequences were then used as input for all further analyses. Clean sequences were analyzed using the ARG-OAP v2.0 pipeline [20] to explore the diversity and abundance of different types and subtypes of ARGs from nine metagenomic datasets in this study. ARG types and subtypes were annotated using default criteria [21]. Metagenomics rapid annotation subsystem technology (MG-RAST) was used to identify bacterial taxonomic classification [22], and a column chart comparing the relative abundance of each class was generated. All clean data were also searched for MGEs against an offline database, including integrons, gene cassettes, insertion sequences and plasmids, downloaded from the INTEGRALL and NCBI RefSeq databases [6,23]. If the nucleotide sequence identity of the best BLASTn hit was ≥90% over an alignment of ≥50 bp with an e-value ≤ 1 × 10^−5^, it was annotated as an integron, insertion sequence or gene cassette. For plasmid annotation, the sequence identity of the BLASTn hit was ≥ 95% with an alignment length ≥ 90 bp [24,25]. Relative abundances of MGEs were calculated using plasmid- and integron-like reads per total metagenomic sequencing read. All statistical analyses were conducted with R software. The correlation coefficient was calculated using Spearman’s rank correlation coefficient method, applying a statistical test with r > 0.6 and *p* < 0.05.

## 3. Results and Discussion

### 3.1. Diversity and Occurrence of ARGs from Metagenomic Analysis

**Figure 1:**
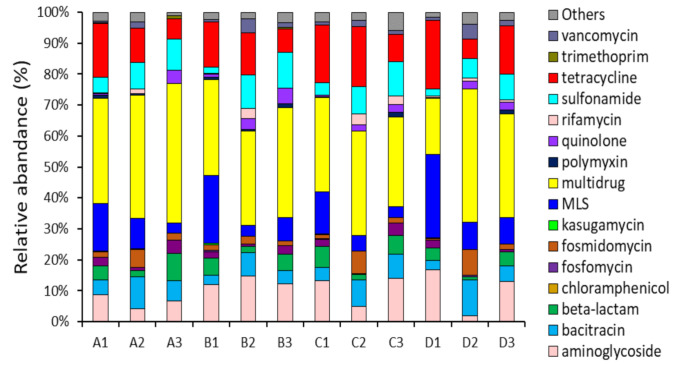
Relative abundance of antibiotic resistance gene (ARG) types in the small (A, B) and large (C, D) WWTP samples. A1, B1, and C1 indicate influent samples, A2, B2, and C2 indicate activated sludge samples, and A3, B3, C3 indicate effluent samples.

Metagenomics analysis revealed that 152 ARG subtypes belonging to 18 ARG types were identified in the small-scale WWTP samples, while 234 ARG subtypes belonging to 21 ARG types were identified in the large-scale WWTP samples. This could be also due to the different number of inhabitants and equivalent of the scale (Table 1). The total abundance of ARGs ranged from 0.38 to 133.07 ppm in small-scale WWTPs and from 2.94 to 145.57 ppm in large-scale WWTPs. As shown in Figure 1, similar types of ARGs, such as aminoglycosides, bacitracin, beta-lactams, multidrug resistance, MLS (macrolide–lincosamide–streptogramin), sulfonamide, and tetracycline resistance genes, were detected between small and large WWTPs, despite differences in their treatment capacities. These abundant ARGs are usually associated with antibiotics used extensively in human or veterinary medicine, including as growth promoters [1,26]. Genes for resistance to MLS, multidrugs, and tetracycline were represented by 70% of all wastewater treatment processes (influent, activated sludge, effluent). Mostly, the abundance and diversity of the detectable ARGs in influent (1.59–145.57 ppm) were significantly higher than those in activated sludge (4.81–38.79 ppm) and effluent (0.38–26.39 ppm), indicating that the biological wastewater treatment process could decrease the ARGs effectively. However, the abundances of multidrug resistance genes were still high, with a range of 21.47 to 26.39 ppm after the treatment process. Interestingly, some resistance genes, such as tetracycline and sulfonamide resistance genes, usually decreased in the effluent during the treatment process in small-scale WWTPs (A and B) but increased in large-scale WWTPs (C and D).

Generally, the removal efficiencies for all WWTP samples were approximately 90% after the treatment process, which may have led to a significant reduction in ARG detection in the effluent samples. The removal of antibiotics is attributed to biodegradation and biosorption onto activated sludge [27,28,29]. In addition, the MLE anoxic/aerobic tanks in the activated sludge process may not provide good conditions for the proliferation of ARG-carrying bacteria; hence, the replication and dissemination of ARGs were not favored [6,30]. However, it is of concern that tetracycline and sulfonamide resistance genes were still high in the effluent because these resistance genes can persist under harsh environmental conditions, which may potentially transfer antibiotic resistance to bacteria via HGT (horizontal gene transfer) [21,31].

**Figure 2:**
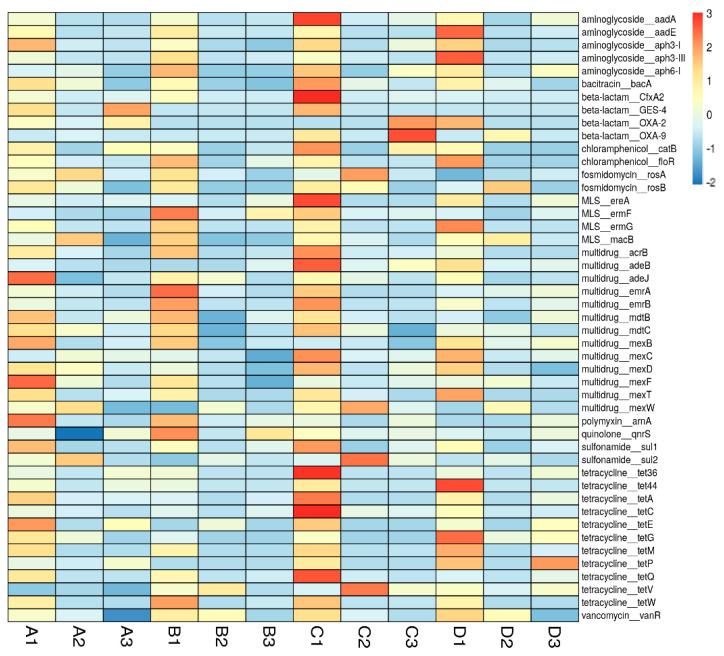
Heatmap of the relative abundance of ARG subtypes (top 50 most abundant) in the small (A, B) and large (C, D) WWTP samples. A1, B1, and C1 indicate influent samples, A2, B2, and C2 indicate activated sludge samples, and A3, B3, C3 indicate effluent samples.

Considering the relative abundance from heatmap analysis, *tet* genes, *OXA* genes, *mex* genes, *mdt* genes, and *sul* genes were the dominant ARGs in the activated sludge samples (Figure 2). However, *aad* genes, *ere* genes, *erm genes*, *mdt* genes, *sul* genes, and *tet* genes were relatively dominant subtypes in the effluent samples. Tetracycline antibiotics are adsorbed by activated sludge flocs or biofilms and are largely concentrated in sludge at levels as high as 0.1–1 mg/L [32,33,34]. The *tet*M gene is commonly localized on a conjugative transposon, Tn916, with a broad host range [35], possibly explaining the observed diversity of the *tet*M-carrying taxa in effluents. TetQ is normally associated with conjugative elements [36], whereas *tet*W has been found to be associated with a conjugative transposon potentially transferred into other genera [37]. MLS also tends to be hydrolyzed or sorbed. Because of the strong sorption of tetracycline and MLS antibiotics, their mobility in the environment may be facilitated by transport with wastewater [38]. Notably, adsorption is one of the major reasons for the development of ARBs along with HGT [12]. *Erm* resistance genes can easily be transferred from one host to another [39] since they are usually acquired and associated with mobile elements, such as plasmids [40] and transposons. Aminoglycoside (*aad*A, *aad*A1 and *aad*A2) and beta-lactam (blaVEM and blaOXA) resistance genes are frequently detected ARGs in WWTPs and are closely related to antibiotics important for human infection treatment or veterinary usage [21]. These characteristics possibly explain why tetracycline, MLS, sulfonamide and multidrug resistance genes were mostly high in abundance in the 12 metagenomes.

**Figure 3:**
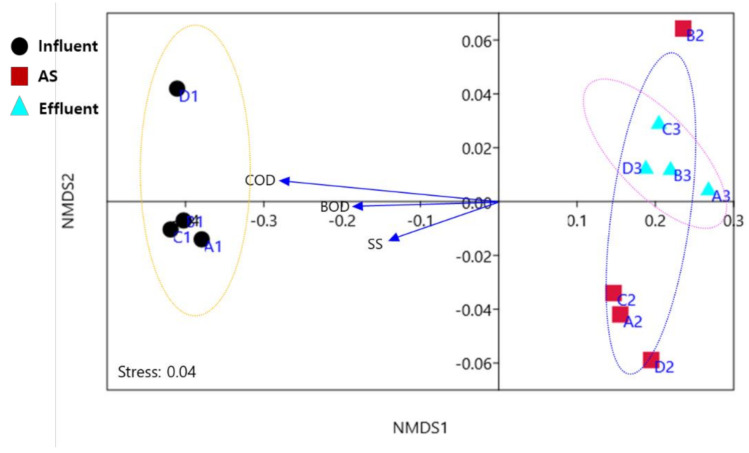
Nonmetric multidimensional scaling (NMDS) of ARG distribution patterns between small (A, B) and large (C, D) WWTP samples. A1, B1, and C1 indicate influent samples, A2, B2, and C2 indicate activated sludge samples, and A3, B3, C3 indicate effluent samples. Stress is a nonnegative number, representing the credibility of the cluster results. A stress value <0.3 indicates high confidence. Blue arrows indicate significant positive correlation parameters (r > 0.6, *p* < 0.05).

As such, the NMDS (nonmetric multidimensional scaling) results showed that the ARG subtypes were primarily influenced by the wastewater treatment process, not the WWTP scale (Figure 3). Contrary to expectation, sample types and origins were generally clustered together. Although physiochemical parameters play an important role in ARG prevalence and dissemination, it remains unknown whether ARGs and physiochemical parameters are significantly correlated between large- and small-scale WWTPs. In the present study, correlation analysis was carried out to understand specific relationships according to WWTP scales. However, there were no significant differences between large- and small-scale WWTPs. Physiochemical parameters such as BOD, COD and SS were positively correlated (*p* < 0.05) with the majority of ARGs in WWTP influents, while negative correlations were observed in effluents. Previous studies reported that pH, COD, and TN/P were positively correlated with ARG abundance in both influent and effluent from municipal WWTPs, and they may be important factors affecting the bacterial community during the treatment process [41,42]. Because raw influent wastewater contains massive amounts of organics and excess nutrients compared to effluent, potentially providing selective pressure for ARB and ARGs [6,12], physiochemical parameters may be positively correlated with major ARG types. Although physiochemical parameters were different according to large and small scale (Table 1), there was no statistically significant difference (*p* > 0.05) between them, and the overall treatment performance (removal rate from influent to effluent) was not quite different. These results suggested that ARG abundance may not be affected by the WWTP scale, but could be affected by influent characteristics in WWTPs. Some previous studies have shown that selected physiochemical parameters are often weakly correlated with ARG abundance in WWTPs, and abundance of ARGs does not rely on operation conditions such as temperature, HRT, SRT and MLSS [41,42,43,44]. Therefore, more studies are required to resolve and determine the underlying physicochemical, biological, and operating drivers for the dissemination of ARG patterns between large and small WWTPs.

### 3.2. Bacterial Community Difference between the Large and Small WWTPs

**Figure 4:**
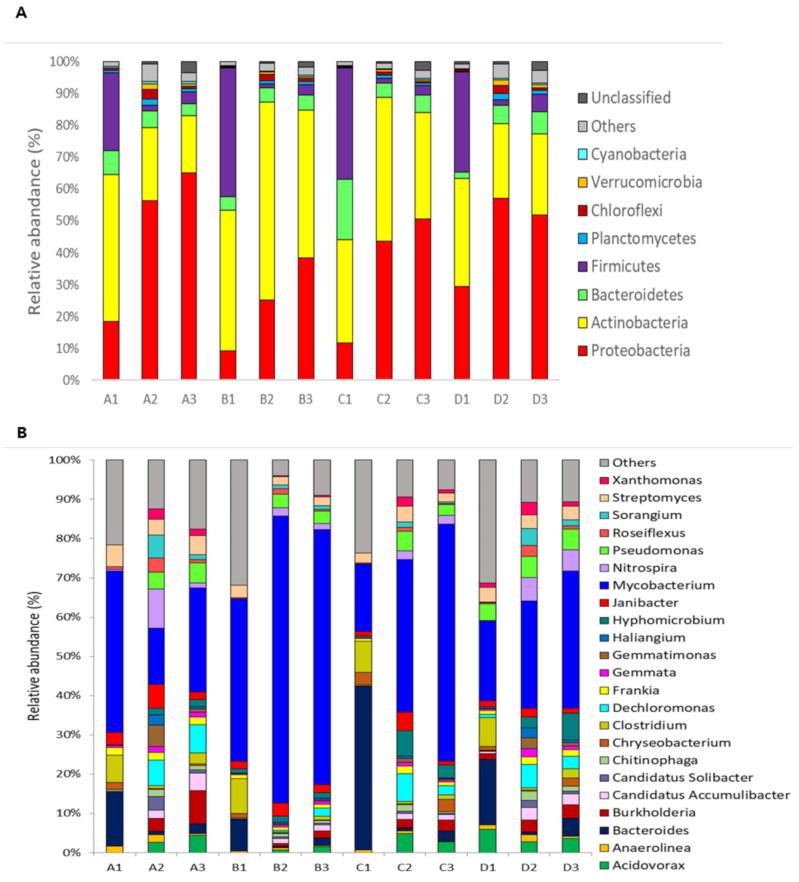
Relative abundance and taxonomic identification of bacteria at the phylum (**A**) and genus levels (**B**) in the small (A, B) and large (C, D) WWTP samples. A1, B1, and C1 indicate influent samples, A2, B2, and C2 indicate activated sludge samples, and A3, B3, C3 indicate effluent samples. Samples with a relative abundance <1% were classified as “others”.

A total of 13 bacterial phyla were identified among all WWTP samples. The profile of bacteria at the phylum level showed that Proteobacteria and Actinobacteria were the most dominant bacteria (Figure 4a). *Proteobacteria* accounted for 10.34% to 20.86% of the bacterial community in the influent, while abundances were significantly higher, ranging from 30.32% to 70.12%, in the activated sludge and effluent from all samples (*p* < 0.05). At the same time, the proportions of *Firmicutes* significantly decreased from the influent (28.86–39.64%) to the activated sludge (3.54–4.21%) and effluent (6.84–9.03%). Among the *Proteobacteria, β-proteobacteria* was the most predominant with a range of 30.9~50.45% among all types of samples between large and small WWTPs, followed by *α-proteobacteria* for 23.8~43.8%, *γ-proteobacteria* for 13.1~23.6%, and *δ-proteobacteria* for 3.7~10.2%. These characteristics were not significantly different between large and small WWTPs. This result is similar to previous results of bacterial communities in most activated sludge studies [6,45], in which *Proteobacteria* was also the most dominant community. *Proteobacteria* are generally known to have an important role in the metabolic capacity of degrading organic pollutants in bioreactors, such as nitrogen, phosphorus, and aromatic compounds [45,46]. *β*- and *γ-Proteobacteria* are abundant and degrade nutrients such as N, and *P* in activated sludge of denitrifying reactors [6,45,46]. *α-Proteobacteria*, nitrite-oxidizing bacteria, and denitrifying capacity were also previously recognized as important members of activated sludge microbial communities [6,45]. *Actinobacteria* play a role in enhanced biological phosphorus removal systems [47], and high abundances of *Actinobacteria* were also reported in previous WWTP systems [48,49].

The most dominant genera also showed similar patterns among sample types (Figure 4b). *Bacteroides, Mycobacterium*, and *Clostridium* were represented by 60~70% of all influent samples. Although *Mycobacterium* (20~60%) still represented the major genera between all activated sludge and effluent samples, shifts in community composition were observed. *Burkholderia, Dechloromonas, Nitrospira, and Pseudomonas* were the major dominant genera in activated sludge and effluent samples regardless of the WWTP scale. These genera are usually detected with high abundance in activated sludge processes in global WWTPs [45]. In fact, members of *Mycobacterium* are among the foaming bacteria in activated sludge due to their high hydrophobic cell surface and enrichment in the foam [50]. Therefore, *Mycobacterium* is known to have good degradation capabilities for aromatic hydrocarbons and nitrogen-containing heterocycles [50]. *Nitrospira* bacteria are the key nitrite oxidizers in sewage treatment plants [51,52]. The *Dechloromonas* and *Burkholderia* genera are known to have an important role in denitrification and the ability to metabolize aromatic compounds [53,54,55]. Among the dominant genera, *Mycobacterium* and *Pseudomonas* are opportunistic pathogen-associated genera, and their detectable levels in effluents may pose problems in receiving waters or may enable regrowth in storage tanks.

The results of this study suggest that the microbial communities are generally different according to the capacity of WWTPs, without some exceptions, such as the dominance of *Mycrobacterium* genera between large- and small-scale WWTPs. This is consistent with previous studies [10,56,57]. Although there are many reasons, similar fitness levels of the microbial community from the influent wastewater community may fit the bioreactor despite different WWTP scales. If specific niches are dominant, such as *Mycobacterium*, and the dispersal rate is not too high, influent wastewater communities may be assembled in bioreactors.

### 3.3. Variation of the Abundance of MGE

**Figure 5:**
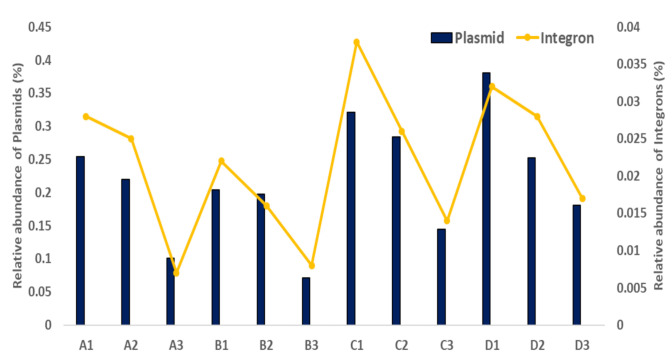
Relative abundance of mobile genetic elements, including plasmids and integrons, in the small (A, B) and large (C, D) WWTP samples. A1, B1, and C1 indicate influent samples, A2, B2, and C2 indicate activated sludge samples, and A3, B3, C3 indicate effluent samples.

As shown Figure 5, the alignment of all the reads to known plasmids showed that large-scale WWTP samples had the highest abundance of all plasmids, which was 1.5–2.0-fold higher than that of the small-scale WWTP samples. In the total numbers of the C and D WWTP samples, known plasmid copies encompassed a range of 0.58 ± 0.16. In contrast, the relative abundance of known plasmids in the A and B WWTP samples was observed to decrease to low levels of 0.33 ± 0.05. Alignment against known integrons showed approximately 1.5-fold and 3-fold higher relative abundances of integron-associated integrases in the large-scale WWTP samples (0.03–0.04%) than in the small-scale WWTP samples (0.008–0.02%) (Figure 5). The class 1 integronase gene *intI1* was predominant in all samples. More types of integrase genes were detected as *intI*, *intI3*, and unknown integrase genes, and their encoding gene cassettes were also detected in this study (data not shown).

The ARG diversity and abundance of both large- and small-scale WWTPs showed a strong correlation with those of the plasmids, showing R^2^ values of 0.66 to 0.84, respectively (Table 2). Considering the different ARG profiles of each sample, the similarity in diversity indices for the parameters of antibiotic resistance suggests that plasmids can aid the persistence of ARGs under different environmental conditions. Plasmids conferring antibiotic resistance are known to be stable in the environment even without antibiotic selection pressures [12,17,58]. In addition, a strong correlation between ARG and integron abundance and diversity, showing an R^2^ value of 0.72 to 0.90, was also detected in this study (Table 2). Integrons are suggested to contribute to the exchange and incorporation of ARGs, resulting in the proliferation of bacterial antibiotic resistance in WWTPs [12,17,58].

HGT via MGEs easily occurs in niches with high biomass and extracellular stress, such as sediments [59], activated sludge [60], and anaerobic digestion [61]. Previous studies reported that *tet* and *sul* resistance genes have a strong association with MGEs to move from one species to another [21]. *tet* resistance genes are usually highly abundant and known to be transferred among bacteria into the receiving environment via HGT [62]. Considering the transfer mechanisms of sulfamethoxazole, *sul1* is carried on *intI,* but *sul1* has been detected on a broad range of host plasmids; this could lead to widespread detection of *sul1* in the wastewater environment [63,64]. The *intI1* gene had intensive connections with multiple ARGs, which can potentially confer resistance to multiple classes of antibiotics. This result was also found in previous studies [8,31,58], thereby suggesting that integrons have widespread coexistence of the same or very similar ARG clusters in various environments because integrons can capture multiple gene cassettes to facilitate the coexistence of ARGs [12,58].

## 4. Conclusions

This study provides a baseline for the different types of antibiotic resistomes and MGEs between small- and large-scale WWTPs. As expected, large-capacity WWTPs had a relatively higher abundance and occurrence of ARGs and MGEs than small-scale WWTPs due to the high input of organic compounds and biomass, which provide favorable environments for the proliferation of ARGs. However, similar taxa structures between large- and small-scale WWTPs assume that specific WWTP niches correspond to regional environmental conditions. Therefore, more studies should be carried out to understand bacterial taxa according to the WWTP scale and their occurrence trends to control the potential relationship between ARG emergence and removal in WWTPs in the future. In addition, the actual relationship between the bacterial community and HGT according to the WWTP scale should be determined in future studies.

## Figures and Tables

**Table 1:** Quality parameters of wastewater samples in effluent.

Category		A	B	C	D
Influent type		Domestic sewage and pretreated industrial wastewater			
Processing Capacity (m^3^/day)		9.5 × 10^4^	4.0 × 10^4^	34.0 × 10^4^	45.2 × 10^4^
Reactor process		KSBNR	DNR	MLE	MLE
Influent wastewater quality	Flow rate (m^3^/days)	42,203	27,124	333,427	357,523
	BOD (mg/L)	63.2 ± 5.35	131.8 ± 4.38	68.8 ± 2.37	84.9 ± 5.37
	COD (mg/L)	130.3 ± 5.75	184.1 ± 25.0	140.8 ± 12.8	178.3 ± 23.1
	SS (mg/L)	126.9 ± 6.41	227.8 ± 29.96	183.6 ± 6.74	182.1 ± 5.77
	T-N (mg/L)	32.0 ± 2.62	57.1 ± 3.39	36.4 ± 1.07	45.5 ± 2.41
	T-P (mg/L)	3.6 ± 0.24	5.8 ± 0.46	3.8 ± 0.064	4.6 ± 0.23
	pH	7.3	7.0	7.3	7.3
Effluent wastewater quality	Flow rate (m^3^/days)	41,111	23,199	303,596	284,540
	BOD (mg/L)	2.9 ± 1.4	6.6 ± 3.2	2.7 ± 0.5	6.5 ± 0.6
	COD (mg/L)	7.9 ± 4.0	16.9 ± 6.3	9.7 ± 3.2	11.2 ± 2.4
	SS (mg/L)	3.2 ± 1.8	4.9 ± 2.1	2.0 ± 0.3	2.8 ± 0.5
	T-N (mg/L)	8.642 ± 2.145	3.948 ± 2.136	9.611 ± 0.526	7.496 ± 2.048
	T-P (mg/L)	1.064 ± 0.037	0.169 ± 0.016	0.419 ± 0.16	0.712 ± 0.026
	pH	6.5	6.7	6.6	7.0
MLSS (mg/L)		2200	1975	2600	2220
HRT (hours)		11	28	14	14.4
SRT (days)		17	7.9	10–15	18.6

**Table 2:** The correlation between antibiotic resistance genes (ARGs) and mobile genetic elements (MGEs).

	MGEs		Integrons		Plasmids	
			Relative Abundance ^1^	Diversity ^2^	Relative Abundance ^1^	Diversity ^2^
Large scale WWTP	ARGs	Relative Abundance ^1^	0.90 **	0.86 **	0.84 *	0.78 *
		Diversity ^2^	0.72 *	0.82 *	0.72 *	0.72 *
Small scale WWTP	ARGs	Relative Abundance ^1^	0.86 **	0.82 **	0.76 *	0.72 *
		Diversity ^2^	0.76 *	0.78 *	0.66 *	0.68 *

* Correlation is significant (*p* < 0.05); **: correlation is highly significant (*p* < 0.01). ^1^ Relative abundance: the portion of ARG and MGE-like sequences in total metagenomic sequences. ^2^ Diversity: the number of annotated ARGs and MGE types.

## Data Availability

The data presented in this study are available on request from the corresponding author.

## Abbreviations

ARG	antibiotic resistance gene
ARB	antibiotic resistant bacteria
AS	activated sludge
DNR	Daewoo nutrient removal
HGT	horizontal gene transfer
KSBNR	Kist Shinwon biological nutrient removal
MGEs	mobile genetic elements
MLE	modified Ludzack–Ettinger
MLS	macrolide–lincosamide–streptogramin
NMDS	nonmetric multidimensional scaling
qPCR	quantitative real time PCR
WWTP	wastewater treatment plant

## References

[1] World Health Organization 2017 WHO, Global Antimicrobial Resistance Surveillance System (GLASS) Report: Early Implementation 2016–2017 (Geneva, 2017). www.who.int/glass/resources/publications/early-implementation-report/en/.

[2] Ma X., Zhang Q., Zhu Q., Liu W., Chen Y., Qiu R., Wang B., Yang Z., Li H., Lin Y. (2015). A Robust CRISPR/Cas9 System for Convenient, High-Efficiency Multiplex genome editing in Monocot and Dicot Plants. Mol. Plant.

[3] Tiedje J.M., Fang W., Manaia C.M., Virta M., Sheng H., Liping M.A., Tong Z., Edward T. (2019). Antibiotic resistance genes in the Human-Impacted environment: A One Health Perspective. Pedosphere.

[4] Marti E., Variatza E., Balcazar J.L. (2014). The role of aquatic ecosystems as reservoirs of antibiotic resistance. Trends Microbiol..

[5] Yang Y., Li Z., Song W., Du L., Ye C., Zhao B., Liu W., Deng D., Pan Y., Lin H. (2019). Metagenomic insights into the abundance and composition of resistance genes in aquatic environments: Influence of stratification and geography. Environ. Int..

[6] Yoo K., Yoo H., Lee J., Choi E., Park J. (2020). Exploring the antibiotic resistome in activated sludge and anaerobic digestion sludge in an urban wastewater treatment plant via metagenomic analysis. J. Microbiol..

[7] Fresia P., Antelo V., Salazar C., Giménez M., D’Alessandro B., Afshinnekoo E., Mason C., Gonnet G.H., Iraola G. (2019). Urban metagenomics uncover antibiotic resistance reservoirs in coastal beach and sewage waters. Microbiome.

[8] Guo J., Li J., Chen H., Bond P.L., Yuan Z. (2017). Metagenomic analysis reveals wastewater treatment plants as hotspots of antibiotic resistance genes and mobile genetic elements. Water Res..

[9] Gupta S.K., Shin H., Han D., Hur H.G., Unno T. (2018). Metagenomic analysis reveals the prevalence and persistence of antibiotic-and heavy metal-resistance genes in wastewater treatment plant. J. Microbiol..

[10] Kim Y.K., Yoo K., Kim M.S., Han I., Lee M., Kang B.R., Lee T.K., Park J. (2019). The capacity of wastewater treatment plants drives bacterial community structure and its assembly. Sci. Rep..

[11] Li D., Liu C.M., Luo R., Sadakane K., Lam T.W. (2015). MEGAHIT: An ultra-fast single node solution for large and complex metagenomics assembly via succinct de Bruijn graph. Bioinformatics.

[12] Rizzo L., Manaia C., Merlin C., Schwartz T., Dagot C., Ploy M.C., Michael I., Fatta-Kassinos D. (2013). Urban wastewater treatment plants as hotspots for antibiotic resistant bacteria and genes spread into the environment: A review. Sci. Total Environ..

[13] Czekalski N., Imminger S., Salhi E., Veljkovic M., Kleffel K., Drissner D., Hammes F., Bürgmann H., Gunten U. (2016). Inactivation of antibiotic resistant bacteria and resistance genes by ozone: From laboratory experiments to full-scale wastewater treatment. Environ. Sci. Technol..

[14] Hembach N., Alexander J., Hiller C., Wieland A., Schwartz T. (2019). Dissemination prevention of antibiotic resistant and facultative pathogenic bacteria by ultrafiltration and ozone treatment at an urban wastewater treatment plant. Sci. Rep..

[15] Pärnänen K., Narciso-da-Rocha C., Kneis D., Berendonk T.U., Cacace D., Do T.T., Elpers C., Fatta-Kassinos D., Henriques I., Jaeger T. (2019). Antibiotic resistance in European wastewater treatment plants mirrors the pattern of clinical antibiotic resistance prevalence. Sci. Adv..

[16] Port J.A., Cullen A.C., Wallace J.C., Smith M.N., Faustman E.M. (2014). Metagenomic frameworks for monitoring antibiotic resistance in aquatic environments. Environ. Health Perspect..

[17] Ju F., Li B., Ma L., Wang Y., Huang D., Zhang T. (2016). Antibiotic resistance genes and human bacterial pathogens: Co-occurrence, removal, and enrichment in municipal sewage sludge digesters. Water Res..

[18] Yoo K., Lee T.K., Choi E.J., Yang J., Shukla S.K., Hwang S., Park J. (2017). Molecular approaches for the detection and monitoring of microbial communities in bioaerosols: A review. J. Environ. Sci..

[19] Bolger A.M., Lohse M., Usadel B. (2014). Trimmomatic: A flexible trimmer for Illumina sequence data. Bioinformatics.

[20] Yin X., Jiang X.T., Chai B., Li L., Yang Y., Cole J.R., Tiedje J.M., Zhang T. (2018). ARGs-OAP v2.0 with an expanded SARG database and Hidden Markov Models for enhancement characterization and quantification of antibiotic resistance genes in environmental metagenomes. Bioinformatics.

[21] Li B., Yang Y., Ma L., Ju F., Guo F., Tiedje J.M., Zhang T. (2015). Metagenomic and network analysis reveal wide distribution and co-occurrence of environmental antibiotic resistance genes. ISME J..

[22] Meyer J.R., Kassen R. (2007). The effects of competition and predation on diversification in a model adaptive radiation. Nature.

[23] Moura A., Soares M., Pereira C., Leitão N., Henriques I., Correia A. (2009). INTEGRALL: A database and search engine for integrons, integrases and gene cassettes. Bioinformatics.

[24] Kwietniewska E., Tys J. (2014). Process characteristics, inhibition factors and methane yields of anaerobic digestion process, with particular focus on microalgal biomass fermentation. Renew. Sustain. Energy Rev..

[25] Shi P., Jia S., Zhang X.X., Zhang T., Cheng S., Li A. (2013). Metagenomic insights into chlorination effects on microbial antibiotic resistance in drinking water. Water Res..

[26] Butaye P., Cloeckaert A., Schwarz S. (2003). Mobile genes coding for efflux-mediated antimicrobial resistance in Gram-positive and Gram-negative bacteria. Int. J. Antimicrob. Agents.

[27] Abegglen C., Joss A., McArdell C.S., Fink G., Schlüsener M.P., Ternes T.A., Siegrist H. (2009). The fate of selected micropollutants in a single-house MBR. Water Res..

[28] Dorival-Garcia N., Zafra-Gomez A., Navalon A., Gonzalez-Lopez J., Hontoria E., Vilchez J.L. (2013). Removal and degradation characteristics of quinolone antibiotics in laboratory-scale activated sludge reactors under aerobic, nitrifying and anoxic conditions. J. Environ. Manag..

[29] Le-Minh N., Khan S.J., Drewes J.E., Stuetz R.M. (2010). Fate of antibiotics during municipal water recycling treatment processes. Water Res..

[30] Ahmed M.B., Zhou J.L., Ngo H.H., Guo W. (2015). Adsorptive removal of antibiotics from water and wastewater: Progress and challenges. Sci. Total Environ..

[31] Wang Z., Zhang X., Huang K., Miao Y., Shi P., Liu B., Long C., Li A. (2013). Metagenomic Profiling of Antibiotic Resistance Genes and Mobile Genetic Elements in a Tannery Wastewater Treatment Plant. PLoS ONE.

[32] Berendonk T.U., Manaia C.M., Merlin C., Fatta-Kassinos D., Cytryn E., Walsh F., Bürgmann H., Sørum H., Norström M., Pons M.-N. (2015). Tackling antibiotic resistance: The environmental framework. Nat. Rev. Microbiol..

[33] Li B., Zhang T. (2011). Mass flows and removal of antibiotics in two municipal wastewater treatment plants. Chemosphere.

[34] Zhang Y., Geng J., Ma H., Ren H., Xu K., Ding L. (2016). Characterization of microbial community and antibiotic resistance genes in activated sludge under tetracycline and sulfamethoxazole selection pressure. Sci. Total Environ..

[35] Roberts A.P., Mullany P. (2009). A modular master on the move: The Tn916 family of mobile genetic elements. Trend Microbiol..

[36] Chopra I., Roberts M. (2001). Tetracycline Antibiotics: Mode of Action, Applications, Molecular Biology, and Epidemiology of Bacterial Resistance. Microbiol. Mol. Biol. Rev..

[37] Kazimierczak K.A., Flint H.J., Scott K.P. (2006). Comparative Analysis of Sequence Flanking *tet*(W) Resistance Genes in Multiple Species of Gut Bacteria. Antimicrob. Agents Chemother..

[38] Kolz A.C., Ong S.K., Moorman T.B. (2005). Sorption of tylosin onto swine manure. Chemosphere.

[39] Roberts M.C. (2004). Resistance to macrolide, lincosamide, streptogramin, ketolide, and oxazolidinone antibiotics. Mol. Biotechnol..

[40] Liu M., Zhang Y., Yang M., Tian Z., Ren L., Zhang S. (2012). Abundance and distribution of tetracycline resistance genes and mobile elements in an oxytetracycline production wastewater treatment system. Environ. Sci. Technol..

[41] Harnisz M., Kiedrzyńska E., Kiedrzyński M., Korzeniewska E., Czatzkowska M., Koniuszewska I., Jóźwik A., Szklarek S., Niestępski S., Zalewski M. (2020). The impact of WWTP size and sampling season on the prevalence of antibiotic resistance genes in wastewater and the river system. Sci. Total Environ..

[42] Yang Y., Liu Z., Xing S., Liao X. (2019). The correlation between antibiotic resistance gene abundance and microbial community resistance in pig farm wastewater and surrounding rivers. Ecotoxicol. Environ. Saf..

[43] Laht M., Karkman A., Voolaid V., Ritz C., Tenson T., Virta M., Kisand V. (2014). Abundances of Tetracycline, Sulphonamide and beta-lactam antibiotic resistance genes in conventional wastewater treatment plants (WWTPs) with different waste load. PLoS ONE.

[44] Sabri N.A., Schmitt H., Van der Zaan B., Gerritsen H.W., Zuidema T., Rijnaarts H.H.M., Langenhoff A.A.M. (2018). Prevalence of antibiotics and antibiotic resistance genes in a wastewater effluent-receiving river in the Netherlands. J. Environ. Chem. Eng..

[45] Wagner M., Loy A. (2002). Bacterial community composition and function in sewage treatment systems. Curr. Opin. Biotechnol..

[46] Yang Y., Li B., Zou S., Fang H., Zhang T. (2014). Fate of antibiotic resistance genes in sewage treatment plant revealed by metagenomic approach. Water Res..

[47] Seviour R.J., Kragelund C., Kong Y., Eales K., Nielsen J.L., Nielsen P.H. (2008). Ecophysiology of the Actinobacteria in activated sludge systems. Antonie Leeuwenhoek..

[48] Schmid M., Thill A., Purkhold U., Walcher M., Bottero J.Y., Ginestet P., Nielsen P.H., Wuertz S., Wagner M. (2003). Characterization of activated sludge flocs by confocal laser scanning microscopy and image analysis. Water Res..

[49] Nielsen P.H., Saunders A.M., Hansen A.A., Larsen P., Nielsen J.L. (2012). Microbial communities involved in enhanced biological phosphorus removal from wastewater—a model system in environmental biotechnology. Curr. Opin. Biotechnol..

[50] Guo F., Zhang T., Li B., Wang Z., Ju F., Liang Y. (2019). Mycobacterial species and their contribution to cholesterol degradation in wastewater treatment plants. Sci. Rep..

[51] Chiellini C., Munz G., Petroni G., Lubello C., Mori G., Verni F., Vannini C. (2013). Characterization and comparison of bacterial communities selected in conventional activated sludge and membrane bioreactor pilot plants: A focus on *Nitrospira* and *Planctomycetes* bacterial *Phyla*. Curr. Microbiol..

[52] Gilbert E.M., Agrawal S., Brunner F., Schwartz T., Horn H., Lackner S. (2014). Response of different nitrospira species to anoxic periods depends on operational DO. Environ. Sci. Technol..

[53] Coates J.D., Chakraborty R., Lack J.G., O’Connor S.M., Cole K.A., Bender K.S., Achenbach L.A. (2001). Anaerobic benzene oxidation coupled to nitrate reduction in pure culture by two strains of Dechloromonas. Nature.

[54] Mao Y., Xia Y., Zhang T. (2013). Characterization of Thauera-dominated hydrogen-oxidizing autotrophic denitrifying microbial communities by using high-throughput sequencing. Bioresour. Technol..

[55] Salinero K.K., Keller K., Feil W.S., Feil H., Trong S., Bartolo G.D., Lapidus A. (2009). Metabolic analysis of the soil microbe Dechloromonas aromatica str. RCB: Indications of a surprisingly complex life-style and cryptic anaerobic pathways for aromatic degradation. BMC Genom..

[56] Ibarbalz F.M., Figuerola E., Erijman L. (2013). Industrial activated sludge exhibit unique bacterial community composition at high taxonomic ranks. Water Res..

[57] Saunders A.M., Albertsen M., Vollertsen J., Nielsen P.H. (2015). The activated sludge ecosystem contains a core community of abundant organisms. ISME J..

[58] Gillings M.R., Gaze W.H., Pruden A., Smalla K., Tiedje J.M., Zhu Y.G. (2015). Using the class 1 integron-integrase gene as a proxy for anthropogenic pollution. ISME J..

[59] Kristiansson E., Fick J., Janzon A., Grabic R., Rutgersson C., Weijdegård B., Söderström H., Larsson D.G.J. (2011). Pyrosequencing of antibiotic-contaminated river sediments reveals high levels of resistance and gene transfer elements. PLoS ONE.

[60] Su J.Q., Wei B., Ou-Yang W.Y., Huang F.Y., Zhao Y., Xu H.J., Zhu Y.G. (2015). Antibiotic Resistome and Its Association with Bacterial Communities during Sewage Sludge Composting. Environ. Sci. Technol..

[61] Christgen B., Scott K., Dolfing J., Head I.M., Curtis T.P. (2015). An evaluation of the performance and economics of membranes and separators in single chamber microbial fuel cells treating domestic wastewater. PLoS ONE.

[62] Agersø Y., Petersen A. (2007). The tetracycline resistance determinant Tet 39 and the sulphonamide resistance gene sulII are common among resistant Acinetobacter spp. isolated from integrated fish farms in Thailand. J. Antimicrob. Chemother..

[63] Pruden A., Pei R., Storteboom H., Carlson K.H. (2006). Antibiotic resistance genes as emerging contaminants: Studies in Northern Colorado. Environ. Sci. Technol..

[64] Chen Q., An X., Li H., Su J., Ma Y., Zho Y.G. (2016). Long-term field application of sewage sludge increases the abundance of antibiotic resistance genes in soil. Environ. Int..

